# The impact of intraoperative diagnosis of spontaneous bacterial peritonitis or bacterascites in liver transplantation

**DOI:** 10.1016/j.clinsp.2026.100993

**Published:** 2026-05-15

**Authors:** Nadielle Q.S. Menezes, Maristela P. Freire, Isabel C.V. Oshiro, Esteban Horacio G. Dominguez, Jairo M. Moreira, Juliana M. Pereira, Wellington Andraus, Luiz Augusto C. D’Albuquerque, Edson Abdala, Alice T.W. Song

**Affiliations:** a Department of Infectious and Parasitic Disorders, Hospital das Clínicas, Faculdade de Medicina, Universidade de São Paulo (HC-FMUSP), São Paulo, SP, Brazil; b Hospital Epidemiology and Infection Control, Hospital das Clínicas, Universidade de São Paulo, São Paulo, SP, Brazil; c Universidad Católica de Cuenca Facultad de Medicina, Gastroenterology Azogues, Cañar, Ecuador; d Division of Liver and Digestive System Organ Transplantation, Department of Gastroenterology, Hospital das Clínicas, Faculdade de Medicina, Universidade de São Paulo (HC-FMUSP), São Paulo, SP, Brazil

**Keywords:** Bacterial infection, Risk factor, Survival, Predictors, Subclinical infection

## Abstract

**Background:**

Bacterial infections can negatively impact post-liver transplant morbidity and mortality, and the impact of incipient spontaneous bacterial peritonitis or bacterascites from intraoperative ascitic fluid is unknown. The present study’s objectives were to analyse the risk factors for post-liver transplant infection and survival within 30-days after transplant.

**Methods:**

This is a single-center retrospective cohort study of liver transplant patients from 2010‒2017, whose ascitic fluid collected during transplant was sent for analysis. Survival analysis using the Kaplan-Meier method and Cox regression in the multivariate analysis was used to determine risk factors for post-transplant infection and survival.

**Results:**

Of 297 patients, 22 had intraoperative SBP, 40 had bacterascites, and 235 had a normal ascitic fluid. 67% were male, median age 53-years, and median MELD score of 24. In multivariate analysis, there was no association between the presence of SBP or the presence of bacterascites and the occurrence of infection or death, and although it was not clinically significant, there was a trend towards worse survival in the bacterascites group. Patients undergoing post-transplant reoperation (OR = 2.28, 95% CI 1.12–4.60, p = 0.022), post-transplant MDR colonization (OR = 4.39, 95% CI 2.61–7.39, p < 0.001), and post-transplant dialysis (OR = 2.20, 95% CI 1.31–3.69, p = 0.003) had a higher rate of infection in the first 30-days after transplantation; pre-transplant MDR colonization, early graft dysfunction and low body mass index were independent predictors of worse survival.

**Conclusions:**

Intraoperative SBP and bacterascites that received targeted treatment did not impact infections nor survival rates up to 30-days after liver transplant, while most episodes were treated. In this population of severe patients with ascites, patients undergoing reoperation, post-transplant MDR colonization and post-transplant dialysis were at higher risk of infection.

## Highlights

• Intraoperative SBP/Bacterascites did not impact infections nor survival rates after liver transplant.
• There was no association between microorganisms isolated from intraoperative ascitic fluid and infectious episodes.
• Patients undergoing reoperation, post-transplant MDRO colonization and post-transplant dialysis were at higher risk of infection.
• Early graft dysfunction and low BMI were identified as risk factors for mortality.

## Introduction

Liver transplantation offers patients with severe liver failure an alternative to increase the recipient's survival and quality of life.^1^ Bacterial infections are the most common cause of infection among patients after liver transplantation, and gram-negative bacilli are the most frequent microorganisms. Bacterial infections primarily involve the surgical site, urinary tract, and bloodstream, and are associated with increased morbidity and mortality rates and graft loss.^2^^,^^3^

In the presence of active and uncontrolled infection, the transplant is usually postponed until there is clinical improvement.^2^ However, there are cases of Spontaneous Bacterial Peritonitis (SBP) or incipient bacterascites incidentally diagnosed from ascitic fluid collected intraoperatively during liver transplantation. In these cases, literature is scarce regarding the impact of these diagnoses in the post-transplant period.^4^ Most studies analyze the impact of pre-transplant diagnosed infections on post-transplant infections and survival outcomes.^5^, ^6^, ^7^, ^8^, ^9^, ^10^, ^11^ However, little is known about the postoperative impact of the subclinical SBP and bacterascites diagnosed intraoperatively on the incidence of post-transplant infections and survival.

This study aims to compare the incidence of bacterial and/or fungal infection and survival in the first 30-days post-liver transplantation in patients with and without intraoperative diagnosed SBP and bacterascites; to evaluate the risk factors for post-liver transplant general infection and surgical site infection within 30-days and survival; and lastly, to compare the microorganisms isolated in the intraoperative ascitic fluid with the causative microorganisms of infections diagnosed within 30-days after transplant.

## Methods

The authors performed a retrospective cohort study, following the STROBE guidelines, consisting of liver transplant patients from the Liver and Digestive Organ Transplant Division at Hospital das Clínicas da Faculdade de Medicina da Universidade de São Paulo in the study period of January 1st 2010, to December 31st 2017. All patients with ascites detected at the time of the first liver transplant, whose material was sent for biochemical and microbiological analysis during the period, were included; as standard, every patient has the fluid collected and sent for analysis during transplant. Patients whose intraoperative analysis was incomplete were excluded. The authors also excluded patients in whom the physiopathology of the presence of ascitic fluid was different from that of cirrhotic patients (fulminant hepatitis, familial polyamyloidosis, polycystic disease); patients with suspected secondary bacterial peritonitis.

Clinical and laboratory data were retrospectively extracted from electronic medical records. Infections were previously identified through active surveillance and review of outpatient records.

### Definitions

Spontaneous bacterial peritonitis, bacterascites and negative ascitic fluid were classified following recommendations from the European Association for the Study of the Liver 2010 Guidelines^12^: No SBP or bacterascites: PMN count is <250 per/mm^3^ and the ascitic fluid culture is negative; SBP: PMN count is greater than 250 per/mm^3^; bacterascites: positive culture with PMN count <250 per/mm^3^; secondary bacterial peritonitis: peritonitis due to perforation or inflammation of an intra-abdominal organ. The criteria used to identify healthcare–associated infections were those of the United States National Healthcare Safety Network^13^; Colonization was defined as carbapenem-resistant gram-negative bacteria or vancomycin-resistant enterococci isolated in surveillance cultures or clinical specimens with no evidence of infection. Early graft dysfunction was classified according to Olthoff,^14^ defined as the presence of one or more of the following postoperative laboratory tests that reflect liver injury and function: total bilirubin ≥10 mg/dL on the seventh day after transplantation; INR ≥ 1.6 on the seventh day after transplantation; alanine/aspartate aminotransferase > 2000 IU/L in the first seven days post-transplantation.

### Treatment protocols

Standard care includes prophylaxis for SBP in cases of previous SBP or ascitic fluid protein < 1.5 mg/dL with norfloxacin 400 mg daily, and in cases of upper gastrointestinal bleeding, ceftriaxone 1 g daily for seven days. In the presence of intraoperative ascites, ascitic fluid is sent for PMN count and culture. Standard surgical prophylaxis includes ampicillin and cefotaxime for 24 h, or ampicillin and amikacin after 2015 in selected cases (colonization by Multidrug Resistant Microorganism – MDR – gram-negatives, MELD > 24, pre-transplant dialysis, use of broad-spectrum antimicrobials 30-days prior to transplantation). Usual immunosuppression includes intraoperative methylprednisolone or basiliximab in the presence of risk factors for post-transplant renal failure, and tacrolimus with or without mycophenolate sodium in addition to prednisone. Surveillance for colonization by multidrug-resistant microorganisms is performed upon admission, and weekly in the ward and in the ICU for Vancomycin-Resistant Enterococci (VRE), carbapenem-resistant *Acinetobacter baumannii*, and carbapenem-resistant *Klebsiella pneumoniae*.

### Statistical analysis

Quantitative variables were represented by median and interquartile range, and categorical variables were presented in absolute (n) and relative (%) frequencies.

In the statistical analysis, the authors used the Chi-Square test or Fisher’s exact test, as indicated, for categorical variables, whereas the Mann-Whitney test was used for continuous variables. Variables showing a value of p < 0.1 in univariate analysis were included in a multivariate analysis, which was performed using Firth’s penalized logistic regression to reduce small-sample bias and address potential issues of data separation. Variables that then reduced the −2 log-likelihood or showed a value of p < 0.05 were retained in the model. Multicollinearity was tested through the variance inflation factor.

Survival curves were constructed through Kaplan-Meier and the log-rank test was used to detect differences between curves.

The study was approved by the hospital’s Ethics Committee (approval n° 3.687.176). All information from medical records and examination systems was kept confidential, and patient identification was preserved. The Ethics Committee stated that a free and informed consent form was not necessary.

## Results

**Fig. 1:**
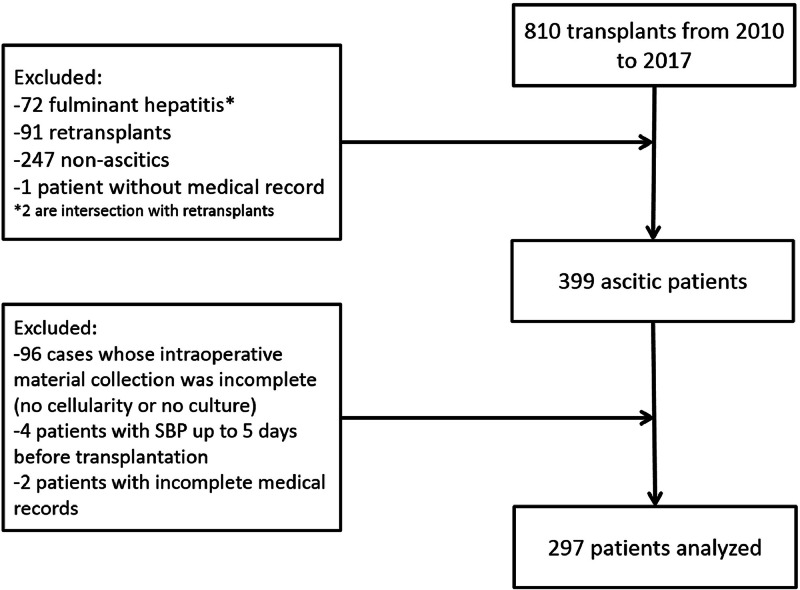
Organizational chart of the patient cohort.

From January 1st, 2010 to December 31st, 2017, 810 liver transplants were performed at the present center. After applying the inclusion and exclusion criteria, 297 patients were included in the study (Fig. 1).

Out of 297 patients, 22 had SBP, 40 had bacterascites and 235 had normal ascitic fluid. None of the patients was characterized with secondary bacterial peritonitis. Table 1 shows the main clinical and demographic data at baseline for patients in both groups. The most common cause of cirrhosis among transplant patients was chronic hepatitis C, followed by alcoholic cirrhosis. There was no difference in the length of hospital stay with a median of 22-days for the groups SBP, bacterascites, and normal ascitic fluid.

**Table 1:** Baseline clinical and demographic data of patients with SBP or bacterascites and intraoperative normal ascitic fluid, 2010‒2017.

	SBP/Bacterascites (n = 62)	Normal ascitic fluid (n = 235)	p
Age	53 (38–61)	54 (46–60)	0.850
Male gender	40 (65%)	158 (67%)	0.680
BMI	25.4 (21.9–29.3)	24.9 (22.8–28.5)	0.790
Functional MELD, median (IQR)	24 (18–32)	24 (16–32)	0.340
Diabetes mellitus	17 (27%)	81 (34%)	0.290
Arterial hypertension	19 (31%)	70 (30%)	0.890
Pre-transplant dialysis	9 (15%)	43 (18%)	0.480
Liver-kidney transplant	4 (6%)	11 (5%)	0.570
Deceased donor	58 (94%)	205 (87%)	0.160
Pre-transplant MDR colonization	19 (31%)	75 (32%)	0.840
Pre-transplant SPB prophylaxis^a^	15 (29%)	72 (38%)	0.270
Previous abdominal surgery^b^	11 (28%)	43 (26%)	0.860
Underlying disease			
HCV	26 (42%)	83 (35%)	0.330
Alcoholic hepatitis	13 (21%)	69 (29%)	0.180
Hepatocarcinoma	18 (30%)	59 (26%)	0.540
Cryptogenic	12 (19%)	36 (15%)	0.440
HBV	3 (5%)	15 (6%)	0.650
Autoimmune	3 (5%)	12 (5%)	0.930
Primary sclerosing cholangitis	3 (5%)	4 (2%)	0.140
Nash	1 (2%)	15 (6%)	0.130
Budd chiari	2 (3%)	5 (2%)	0.610
Primary biliary cirrhosis	0 (0%)	7 (3%)	0.160
Secondary biliary cirrhosis	1 (2%)	2 (1%)	0.590
Caroli's disease	0 (0%)	3 (1%)	0.370
A1 antitrypsin deficiency	0 (0%)	2 (1%)	0.460
Hemochromatosis	0 (0%)	1 (0.5%)	0.600
Wilson	0 (0%)	1 (0.5%)	0.600
Other diagnoses	32 (52%)	91 (39%)	0.060
Indication of LT by special situation			
Ascites	22 (35%)	76 (32%)	0.64
Encephalopathy	8 (13%)	40 (17%)	0.43
Upper gastrointestinal bleeding	12 (19%)	46 (20%)	0.96
SBP	8 (13%)	37(16%)	0.57
Itching	0 (0%)	2 (1%)	0.46
Pre-transplant ICU admission	14 (10%)	15 (10%)	0.98

BMI, Body Mass Index; MELD, Model for End-Stage Liver Disease; IQR, Interquartile Range; MDR, Drug Resistant; SBP, Spontaneous Bacterial Peritonitis; LT, Liver Transplantation; HCV, Hepatitis C Virus; HBV, Hepatitis B Virus; NASH, Nonalcoholic steatohepatitis; ICU, Intensive Care Unit; BMI, Body Mass Index.

a Data from 242 of 297 patients.

b Data from 204 of 297 patients.

In 22 patients with SBP criteria, 3 had a positive culture (*Klebsiella pneumoniae* in one case*; Klebsiella pneumoniae* and *Staphylococcus aureus* in one case*;* and *Staphylococcus warneri* in one case). In the bacterascites group, the most frequently found microorganisms were gram-positive cocci (n = 31, 77% of cases), mainly coagulase-negative *Staphylococcus*, followed by gram-negative bacilli (n = 12, 30%) of which *Klebsiella pneumoniae* (5-microorganisms) was the most frequent. Of the 43 patients with bacterial growth in the intraoperative ascitic fluid, 37 received appropriate antibiotic therapy in the immediate postoperative period for at least 5-days according to susceptibility testing. In these cases, the antibiotic received was retrospectively analyzed by the present study, and it was determined whether or not it covered the agent found in the ascitic fluid based on the interpretation of the antibiogram. The clinical team did not necessarily already know the culture result; the regimens were initiated for various reasons, including laboratory and clinical changes that were judged as a possible infectious process. All patients with SBP but no positive culture were treated with cefotaxime for 5‒7 days post-operatively.

Regarding pre-transplant MDR colonization, 131 of 297 (44%) were already colonized prior to transplantation, and some patients were colonized with more than one MDR species. The most frequent colonizing pre-transplant agent was VRE in 51 of 131 patients (39%), followed by carbapenem-resistant *Klebsiella pneumoniae* (35%). There were 88 of 297 patients (30%) with post-transplant MDR colonization. The most frequent was carbapenem-resistant *Klebsiella pneumoniae* (33%), followed by VRE (29%).

There were 114 patients (38%) with post-transplantation infections in the 30-day period after transplant. There were 63/114 (48%) episodes of organ-space surgical site infections. Catheter-related bloodstream infection was the second most frequent (25 episodes, 19%), followed by pneumonia – 19 episodes (14%).

In surgical site infections, there was a higher prevalence of gram-negative bacilli as causative microorganisms. The most frequent was *Klebsiella pneumoniae*, present in 41 samples (28%), followed by *Acinetobacter baumanii* in 17-episodes (12%), *Enterococcus faecium* in 12 (8%). Sixty-seven (45%) had a multidrug-resistant profile. Among the two patients in whom the microorganism isolated from intraoperative ascitic fluid also caused post-transplant infection, the first had a MELD score of 31 and a recent episode of spontaneous bacterial peritonitis 16-days before transplantation. *Enterobacter cloacae* was isolated intraoperatively, and the patient received imipenem and colistin. Six days after transplant, he developed an organ-space surgical site infection caused by the same organism; antimicrobial therapy was continued for 14-days, and he was discharged after 79-days. The second patient, with a MELD score of 32, had no pre-transplant infection but was colonized with carbapenem-resistant *Klebsiella pneumoniae*. The same organism with an identical susceptibility profile was isolated from intraoperative ascitic fluid and subsequently caused a post-transplant urinary tract infection. He was treated with imipenem and colistin and discharged 29-days after transplantation. Due to the very low number of cases, the authors were unable to include them in the bivariate or multivariate analysis to understand the implications of this finding.

**Fig. 2:**
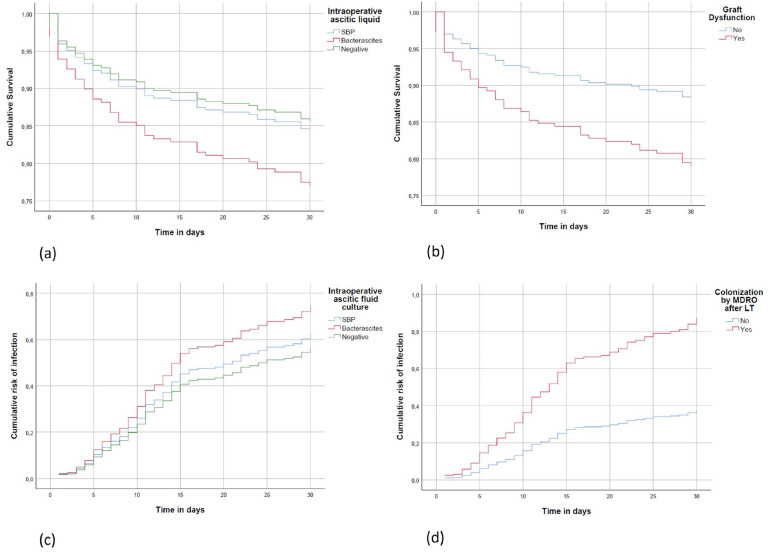
(a) Cumulative 30-day survival curve by intraoperative ascitic fluid culture results, adjusted for pre-transplant MDR colonization, early allograft dysfunction, and body mass index. (b) Cumulative 30-day survival curve by the presence of early graft dysfunction, adjusted for pre-transplant MDR colonization and body mass index. (c) Cumulative risk curve of infection within 30-days after liver transplantation by intraoperative ascitic fluid culture results, adjusted for reoperation, post-transplant MDR colonization, and post- transplant dialysis. (d) Cumulative risk curve of infection within 30-days after liver transplantation by the presence of multidrug-resistant bacterial colonization post-transplant, adjusted for reoperation and post-transplant dialysis.

Bivariate and multivariate analyses of risk factors for post-transplant infection are shown in Table 2, with no impact of the intraoperative diagnosis of SBP or bacterascites. The occurrence of post-transplant reoperation (OR = 2.28, 95% CI 1.12–4.60, p = 0.022), post-transplant MDR colonization (OR = 4.39, 95% CI 2.61–7.39, p < 0.001), and post-transplant dialysis (OR = 2.20, 95% CI 1.31–3.69, p = 0.003) remained independent risk factors for post-transplant infection. Fig. 2 shows the Kaplan-Meier curves for post-transplant infection according to the risk factors. Table 3 describes the bivariate and multivariate analyses for surgical site infections, which were the most frequent in the present study. Diagnosis of hepatocellular carcinoma (OR = 0.49, 95% CI 0.24–0.98, p = 0.05) and colonization by an MDR agent after transplantation (OR = 2.46, 95% CI 1.40–4.33, p = 0.002) were independent risk factors for surgical site infection.

**Table 2:** Univariate analysis and multivariate of risk factors for infection at 30-days post-transplant in liver transplant patients with ascites, 2010‒2017.

	**Post-transplant infection ‒ bivariate analysis**				**Multivariate analysis**		
	**Yes** **(n=132)**	**No** **(n=165)**	**RR (Relative Risk)**	**p**	**OR (Odds Risk)**	**Confidence interval**	**p**
Functional MELD	24 (17–32)	23 (14–31)	‒	0.031	‒	‒	‒
Feminine gender	48 (33%)	51 (33%)	1.00 (0.79–1.26)	1.000	‒	‒	‒
Age	54 (42–60)	54 (46 – 61)	‒	0.468	‒	‒	‒
Diabetes mellitus	48 (33%)	51 (34%)	0.99 (0.78–1.25)	0.968	‒	‒	‒
Arterial hypertension	43 (30%)	46 (30%)	0.98 (0.77–1.25)	0.910	‒	‒	‒
Cirrhosis of alcoholic etiology	45 (55%)	37 (45%)	1.20 (0.91–1.56)	0.173			
Hepatocarcinoma	30 (21%)	46 (31%)	0.78 (0.62–0.99)	0.054	‒	‒	‒
Abdominal surgery prior to LT^a^	19 (35%)	35 (65%)	0.65 (0.39–1.09)	0.105	‒	‒	‒
Antibiotic in the 30-days pre-transplant^b^	43 (52%)	27 (46%)	1.16 (0.79–1.72)	0.434	‒	‒	‒
MDR colonization prior to LT	51 (35%)	33 (22%)	1.43 (1.07–1.92)	0.008			
Pre-transplant infection	62 (44%)	43 (32%)	1.30 (0.99–1.70)	0.430	‒	‒	‒
Pre-transplant dialysis	30 (21%)	22 (14%)	1.26 (0.90–1.77)	0.144	‒	‒	‒
SBP prophylaxis^c^	45 (39%)	38 (33%)	1.14 (0.86–1.51)	0.338			
Pre-transplant ICU admission	12 (9%)	17 (10%)	1.08 (0.71–1.65)	0.713	‒	‒	‒
Length of stay, days	31 (19–48)	16 (11–25)	‒	0.124			
Antibiotic therapy on the day of LT^d^	15 (48%)	16 (52%)	0.98 (0.55–1.72)	0.949	‒	‒	‒
Use of broad-spectrum antibiotics in TF prophylaxis	41 (31%)	47 (28%)	1.13 (0.68–2.41)	0.629	‒	‒	‒
Liver-kidney transplant	9 (6%)	6 (4%)	1.28 (0.68–2.98)	0.393	‒	‒	‒
Surgery time, hours	8 (6–9)	7 (7–9)	‒	0.474	‒	‒	‒
Cold ischemia time, hours	6 (4–7)	6 (5–8)	‒	0.325	‒	‒	‒
Post-TF ICU time, days	7 (5–14)	5 (3–8)	‒	0.038	‒	‒	‒
Intraoperative SBP	12 (9%)	10 (6%)	1.14 (0.71–1.83)	0.554	0.84	(0.31–2.24)	0.724
Corticosteroid bolus^e^	88 (85%)	86 (80%)	1.17 (0.85–1.59)	0.344	‒	‒	‒
Post-transplant CMV infection	17 (12%)	18 (%)	1.00 (0.711–1.41)	0.991	‒	‒	‒
Graft dysfunction (Olthoff)	73 (54%)	65 (47%)	1.14 (0.90–1.45)	0.249	‒	‒	‒
Acute rejection up to 30-days post-transplants	20 (14%)	28 (18%)	0.861 (0.65–1.12)	0.302	‒	‒	‒
Biliary complications up to 30-days after transplantation	7 (5%)	7 (5%)	1.03 (0.60–1.76)	0.907	‒	‒	‒
Vascular complications up to 30-days after transplantation	15 (11%)	28 (12%)	0.977 (0.67–1.40)	0.900	‒	‒	‒
Post transplant reoperation	36 (25%)	17 (11%)	1.73 (1.15–2.61)	0.002	2.28	(1.12–4.60)	0.022
Retransplant	14 (11%)	10 (6%)	1.80 (0.85–3.80)	0.08			
Post-transplant MDR colonization	105 (73%)	53(35%)	2.15 (1.68–2.73)	<0.001	4.39	(2.61–7.39)	<0.001
Antibiotic use > 24-hours post LT	50 (38%)	54 (33%)	1.25 (0.77–2.02)	0.355	‒	‒	‒
Post-transplant dialysis	93 (65%)	55 (36%)	1.77 (1.39–2.24)	<0.001	2.20	(1.31–3.69)	0.003
Antibiotic treament for cultures intraoperative^f^	19 (51%)	8 (22%)	2.3 (0.88–6.23)	0.020	‒	‒	‒
BMI	25 (14–49)	26 (17–38)	‒	0.104			

Data in parentheses: 25‒75 percentiles and percentages. Numerical variables in median and dichotomous in absolute numbers.

MELD, Model for End-Stage Liver Disease; TF, Liver Transplantation; MDR, Multi-Resistant; ICU, Intensive Care Unit; LA, Ascitic Fluid; RR, Relative Risk; CI, Confidence Interval; SBP, Spontaneous Bacterial Peritonitis.

a Data from 204 of 297 patients.

b Data from 131 out of 297 patients.

c Data from 232 out of 297.

d Data from 172 out of 297 patients.

e Data from 212 out of 297.

f Data from 37 out of 43 patients with positive intraoperative culture. This 37 received antibiotic therapy considered effective for the agent in question.

**Table 3:** Univariate analysis and multivariate of risk factors for surgical site infection at 30-days post-transplant in liver transplant patients with ascites, 2010‒2017.

	**Post-transplant infection ‒ bivariate analysis**			**Multivariate analysis**		
	**RR (relative risk)**	**p**		**OR (Odds risk)**	**Confidence interval**	**p**
Functional MELD	‒	**0.05**		‒	‒	‒
Feminine gender	**0.98 (0.85‒1.13)**	**0.78**		‒	‒	‒
Age	‒	0.21		‒	‒	‒
Diabetes mellitus	0.99 (0.86‒1.15)	0.98		‒	‒	‒
Arterial hypertension	1.01 (0.87‒1.17)	0.89		‒	‒	‒
Hepatocarcinoma	0.83 (0.73‒0.94)	0.01	Multiple imputation	0.50	0.25‒0.99	0.05
			Firth regression	0.50	0.25‒1.01–0.50	0.05
Abdominal surgery prior to LT^a^	0.94 (0.80‒1.11)	0.46		‒	‒	‒
Antibiotic in the 30-days pre-transplant^b^	1.05 (0.84‒1.31)	0.67		‒	‒	‒
MDR colonization prior to LT	1.12 (0.95‒1.32)	0.15		‒	‒	‒
Pre-transplant infection	1.06 (0.91‒1.24)	0.44		‒	‒	‒
Pre-transplant dialysis	0.89 (0.78‒1.04)	0.21		‒	‒	‒
SBP prophylaxis^c^	0.99 (0.84‒1.16)	0.88		‒	‒	‒
Pre-transplant ICU admission	0.97 (0.77‒1.22)	0.79		‒	‒	‒
Length of stay, days	‒	0.50		‒	‒	‒
Antibiotic therapy on the day of LT^d^	0.94 (0.77‒1.14)	0.56		‒	‒	‒
Use of broad-spectrum antibiotics in TF prophylaxis	1.02 (0.86‒1.19)	0.86		‒	‒	‒
Liver-kidney transplant	0.90 (0.69‒1.17)	0.77		‒	‒	‒
Surgery time, hours	‒	0.47		‒	‒	‒
Cold ischemia time, hours	‒	0.93		‒	‒	‒
Post-TF ICU time, days	‒	0.99		‒	‒	‒
Intraoperative SBP diagnosis	0.99 (0.84‒1.17)	0.93	Multiple imputation	0.92	0.62‒1.34	0.64
			Firth regression	1.15	0.43‒3.10 – 1.15	0.79
Corticosteroid bolus^e^	1.09 (0.90‒1.32)	0.41		‒	‒	‒
Post-transplant CMV infection	1.24 (0.96‒1.60)	0.05		‒	‒	‒
Graft dysfunction (Olthoff)	1.07 (0.93‒1.23)	0.32		‒	‒	‒
Acute rejection up to 30-days post-transplants	1.13 (0.91‒1.39)	0.22		‒	‒	‒
Biliary complications up to 30-days after transplantation	1.49 (0.80‒2.78)	0.08		‒	‒	‒
Vascular complications up to 30-days after transplantation	1.07 (0.83‒1.39)	0.56		‒	‒	‒
Post-transplant reoperation	1.20 (0.96‒1.49)	0.06	Multiple imputation	1.76	0.92‒3.40	0.09
			Firth regression	1.74	0.91‒3.33 – 1.74	0.09
Retransplant	1.15 (0.80‒1.64)	0.38		‒	‒	‒
Post-transplant MDR colonization	1.28 (1.12‒1.47)	<0.001	Multiple imputation	2.52	1.43‒4.45	0.001
			Firth regression	2.41	1.38‒4.22 – 2.41	0.002
Antibiotic use > 24-hours post LT	0.78 (0.40‒1.55)	0.65		‒	‒	‒
Post-transplant dialysis	1.20 (1.05‒1.38)	0.008		‒	‒	‒
BMI	‒	0.69		‒	‒	‒

Data in parentheses: 25‒75 percentiles and percentages. Numerical variables in median and dichotomous in absolute numbers.

MELD, Model for End-Stage Liver Disease; TF, Liver Transplantation; MDR, Multi-Resistant; ICU, Intensive Care Unit; LA, Ascitic Fluid; RR, Relative Risk; CI, Confidence Interval; SBP, Spontaneous Bacterial Peritonitis.

a Data from 204 of 297 patients.

b Data from 131 out of 297 patients.

c Data from 232 out of 297.

d Data from 172 out of 297 patients.

e Data from 212 out of 297. ^6^ Data from 37 out of 43 patients with positive intraoperative culture. This 37 received antibiotic therapy considered effective for the agent in question.

In the bivariate analysis of mortality within 30-days post-transplantation, risk factors included: abdominal surgery prior to transplantation, pre-transplant MDR colonization, length of stay, corticosteroid bolus, post-transplant cytomegalovirus infection, acute rejection up to 60 days post-transplant, early graft dysfunction, and low BMI (Table 4). In the multivariate analysis, the independent risk factors were pre-transplant MDR colonization (OR = 2.16, 95% CI 1.11–4.22, p = 0.02), early graft dysfunction (OR = 2.33, 95% CI 1.18–4.59, p = 0.02), and low BMI (OR = 1.06, 95% CI 1.00–1.13, p = 0.04).

**Table 4:** Univariate and multivariate analysis of risk factors for death at 30-days post-transplantation in liver transplant patients with ascites, 2010‒2017.

	**Death at 30-days post-transplantation ‒ bivariate analysis**			**Multivariate analysis**			
	**Yes** **(n=57)**	**No** **(n=240)**	**RR (Relative Risk)**	**p**	**RR (Relative Risk)**	**Confidence interval**	**p**
Functional MELD	26 (17–32)	24 (16–32)	‒	0.196	‒	‒	‒
Feminine gender	12 (23%)	87 (36%)	1.10 (0.99–1.21)	0.084	‒	‒	‒
Age	56 (46–62)	54 (45‒60)	‒	0.280	‒	‒	‒
Diabetes mellitus	12 (23%)	87 (35%)	0.90 (0.81–1.00)	0.081	‒	‒	‒
Arterial hypertension	12 (23%)	77 (32%)	0.93 (0.83–1.03)	0.220	‒	‒	‒
Cirrhosis of alcoholic etiology	18 (22%)	64 (78%)	1.14 (0.59–2.18)	0.681	‒	‒	‒
Hepatocarcinoma	10 (20%)	66 (27%)	0.94 (0.84–1.05)	0.341	‒	‒	‒
Abdominal surgery prior to TF^a^	3 (6%)	51 (94%)	0.91 (0.12–0.67)	0.019	‒	‒	‒
Antibiotic in the 30-days pre-transplant^b^	15 (50%)	55 (50%)	1.00 (0.84‒1.19)	0.965	‒	‒	‒
Pre-transplant infection	15 (38%)	90 (38%)	1.00 (0.91‒1.10)	0.939	‒	‒	‒
Pre-transplant MDR colonization	23 (44%)	61 (25%)	1.19 (1.03‒1.37)	0.005	2.16	1.11‒4.22	0.02
Pre-transplant dialysis	12 (23%)	40 (16%)	1.08 (0.92‒1.27)	0.245		‒	‒
SBP prophylaxis^b^	21 (44%)	62 (34%)	1.09 (0.94‒1.26)	0.196	‒	‒	‒
Pre-transplant ICU admission	10 (18%)	19 (8%)	1.16 (0.90‒1.49)	0.150	‒	‒	‒
Length of stay, days	6 (1‒16)	19 (12‒30)	‒	0.001	‒	‒	‒
Antibiotic therapy on the day of LT^c^	11 (30%)	22 (16%)	1.78 (0.98‒3.22)	0.066	‒	‒	‒
Use of broad-spectrum antibiotics in TF prophylaxis	16 (28%)	72(30%)	0.92 (0.55‒1.56)	0.774	‒	‒	‒
Liver-kidney transplant	3 (6%)	12 (5%)	1.02 (0.79‒1.33)	0.826	‒	‒	‒
Surgery time, hours	8 (6‒11)	8 (7‒9)	‒	0.114	‒	‒	‒
Cold ischemia time, hours	6 (5‒8)	6 (5‒8)	‒	0.879	‒	‒	‒
Post-TF ICU time, days	5 (1‒11)	6 (4‒9)	‒	0.120	‒	‒	‒
Intraoperative SBP	7 (12%)	15 (6%)	1.01(0.38‒2.42)	>0.99	0.95	0.60‒1.32	0.74
Corticosteroid bolus	30 (71%)	144 (85%)	0.45 (0.20‒0.99)	0.045	‒	‒	‒
Post-transplant infection	25 (48%)	119 (49%)	1.24 (0.68‒2.23)	0.948	‒	‒	‒
Post-transplant CMV infection	4 (7%)	37 (15%)	0.84 (0.77‒0.92)	0.006	‒	‒	‒
Graft dysfunction (Olthoff)	33 (68%)	105 (47%)	1.16 (1.04–1.30)	0.005	2.33	1.18‒4.59	0.02
Acute rejection to 30-days post-transplants	4 (7%)	45 (18%)	0.85 (0.78‒0.94)	0.020	‒	‒	‒
Biliary complications up to 30-days after transplantation	4 (7%)	14 (6%)	1.24 (0.49‒3.16)	0.732	‒	‒	‒
Vascular complications up to 30-days after transplantation	7 (15%)	39 (17%)	0.96 (0.84‒1.10)	0.644	‒	‒	‒
Post-transplant reoperation	13 (29%)	60 (24%)	0.99 (0.88‒1.11)	0.870	‒	‒	‒
Retransplant	1 (2%)	16 (7%)	0.87 (0.76‒0.99)	0.320			
Post-transplant MDR colonization	24 (14%)	145 (86%)	0.76 (0.42‒1.37)	0.085	‒	‒	‒
Antibiotic use >24 h post LT	17 (30%)	87 (36%)	0.78 (0.47‒1.31)	0.361	‒	‒	‒
Post-transplant dialysis	29 (55%)	119 (48%)	1.05 (0.94‒1.16)	0.346	‒	‒	‒
BMI	26 (14‒49)	33 (19‒41)		0.026	1.06	1.00‒1.13	0.04

Data in parentheses: 25–75 percentiles and percentages. Numerical variables in median and dichotomous in absolute numbers.

TF, Liver Transplantation; MDR, Multi-Resistant; ICU, Intensive Care Unit; LA, Ascitic Fluid; RR, Relative Risk; CI, Confidence Interval; SBP, Spontaneous Bacterial Peritonitis.

a Data from 204 of 297 patients.

b Data from 131 out of 297 patients.

c Data from 172 out of 297 patients MELD: Model for End-Stage Liver Disease.

Survival at 30-days was 88% in the SBP group, 81% in the bacterascites group, and 81% in the normal ascitic fluid group (p = 0.076). The 1-year survival was 73% in the SBP group, 62% in the bacterascites group, and 74% in the normal ascitic fluid group (p = 0.119). The authors see that there is a trend towards worse survival in the bacterascites group compared to patients with normal ascitic fluid, although there is no statistical significance.

## Discussion

This study evaluated the post-transplant impact of intraoperatively diagnosed Spontaneous Bacterial Peritonitis (SBP) or bacterascites, a clinical scenario that has been rarely investigated. The authors found no evidence that these intraoperative findings were associated with increased post-transplant infections or mortality, likely because most patients with positive cultures received appropriate antimicrobial therapy.

Most previous studies assessing the impact of infections on liver transplant outcomes have focused on infections diagnosed before transplantation, ranging from 30-days to one year prior.^5^, ^6^, ^7^, ^8^, ^9^, ^10^, ^11^ The study that most reflects the objectives of this study is the one done by McDonald et al., with no increase in mortality or changes in transplant decision-making among patients with subclinical SBP diagnosed immediately before transplantation.^4^ Although infections associated with clinical instability may increase mortality, transplantation may still be beneficial in stable patients, particularly given MELD score increases during infectious episodes.^15^

In the present study’s cohort, post-transplant reoperation, Multidrug-Resistant (MDR) colonization, and dialysis were independent risk factors for post-transplant infection. Kim et al. reported higher mortality among liver transplant recipients who developed post-transplant vancomycin-resistant *Enterococcus* infections, consistent with this finding that pre-transplant MDR colonization was associated with death.^7^ Freire et al. demonstrated that post-transplant dialysis increased the risk of MDR infections, particularly carbapenem-resistant Enterobacteriaceae, and identified kidney-liver transplantation and MELD >32 as additional risk factors.^16^ Leibovici-Weissman et al. found that multiple infectious episodes, older age, male sex, and higher MELD scores were associated with increased mortality during long-term follow-up.^17^ The high 60-day mortality observed in the present cohort (29% and 19% in both groups) likely reflects the inclusion of high-risk patients with elevated MDR colonization rates.

Although pre-transplant colonization was not an independent predictor of post-transplant infection, the high prevalence of MDR organisms likely reflects frequent hospitalizations and invasive procedures during prolonged waiting-list periods, as previously described.^18^

While Mounzer et al. found no association between Body Mass Index (BMI) and SBP,^8^ the authors observed lower survival among patients with lower BMI. Prior studies have shown that BMI < 18.5 is associated with poorer post-transplant survival and may inadequately reflect nutritional status in end-stage liver disease due to anasarca. .^2^^,^^19^^,^^20^ Additionally, the studied group and others have previously demonstrated an association between higher functional MELD scores and mortality, possibly reflecting the inclusion of more severely ill patients with ascites. Additionally, the studied group and others have previously demonstrated an association between higher functional MELD scores and mortality, possibly reflecting the inclusion of more severely ill patients with ascites.^11^^,^^21^ Early graft dysfunction has also been identified as a predictor of mortality, particularly in the presence of elevated bilirubin and INR values.^22^ Importantly, although it was not clinically significant, there was a trend towards worse survival in the bacterascitis group, and this merits further studies.

There were only 2 cases in which the same microorganism isolated in the ascitic fluid intraoperatively caused an infection (intrabdominal surgical site infection and urinary tract infection), despite treatment. In the first case, there is a possible association of the positive culture and the post-transplant surgical site infection, and in the second case, the patient was colonized by the MDR *Klebsiella pneumoniae* and presented a urinary tract infection by the same microorganism, no conclusions can be drawn. In both cases, the patients were successfully discharged from the hospital.

The authors observed a high prevalence of gram-positive cocci in intraoperative ascitic fluid cultures, with limited impact on post-transplant infections. Similar findings have been reported by Castellote et al. and McDonald et al., who identified coagulase-negative staphylococci as the predominant organisms in bacterascites.^4^^,^^23^ This shift toward gram-positive organisms has been attributed to prolonged quinolone prophylaxis, despite their historical classification as contaminants,^24^^,^^25^ despite being microorganisms classically considered as contaminants. Given prior outbreaks of *Staphylococcus epidermidis* at the center, these isolates were considered clinically significant. Organ-space surgical site infections were the most frequent post-transplant infections, followed by bloodstream infections and pneumonia, consistent with prior studies and likely related to extensive hepatobiliary manipulation during transplantation.^6^^,^^7^^,^^26^^,^^27^

Although not statistically significant, both 60-day (71% vs. 81%, p = 0.076) and 1-year survival (65% vs. 74%, p = 0.119) showed a consistent trend toward worse outcomes in patients with SBP or bacterascites.

The main limitations of the study include the lack of clinical data regarding the immediate pretransplant period in patients who presented with SBP or bacterascites, which precluded conclusions about the clinical presentation despite these diagnoses. Another limitation was the difficulty in differentiating contamination from true bacterascites in cases with gram-positive bacterial growth, although several previous studies have also reported a significant role for gram-positive bacteria in this context. Furthermore, an important limitation is that 37 out of 43 patients (86%) with SBP or bacterascites received antimicrobial therapy targeting the microorganism identified in the intraoperative ascitic fluid; therefore, no conclusions can be drawn regarding whether untreated cases would lead to similar outcomes. In addition, the results should be interpreted with caution, as this cohort comprised severely ill patients with high rates of MDR colonization, and further studies in different populations are needed. The strengths of the study include its originality and the inclusion of variables that are rarely evaluated, such as early graft dysfunction according to Olthoff’s criteria.

## Conclusions

The authors found no difference in the occurrence of bacterial and/or fungal infections within the first 30-days after liver transplantation between patients with intraoperatively diagnosed SBP, subclinical bacterascites, and those with normal ascitic fluid, although the majority of cases received targeted antimicrobials after transplant. Moreover, there was no association between microorganisms isolated from intraoperative ascitic fluid and infectious episodes diagnosed within 30-days after transplantation. A higher incidence of infection within the first 30-days post-transplant was observed among patients who underwent post-transplant reoperation, had post-transplant MDR colonization, or required post-transplant dialysis. With respect to survival, no differences were observed between the two groups at 30-days or 1-year after transplantation. Early graft dysfunction and low BMI were identified as risk factors for mortality. There was no impact on the incidence of post-transplant infection or on survival among liver transplant candidates who presented with either subclinical spontaneous bacterial peritonitis or bacterascites and received targeted antimicrobial therapy, and further studies should be performed regarding the finding of the trend towards worse survival in the bacterascites group.

**Abbreviations**
BMI: Body Mass Index
CI: Confidence Interval
ICU: Intensive Care Unit
INR: International Normalized Ratio
IU: International Units
L: Liter
MDR: Multi-Resistant
MELD: Model for End-Stage Liver Disease
Mg: Milligram
Mm: Milliliter
PMN: Polymorphonuclear
RR: Relative Risk
SBP: Spontaneous Bacterial Peritonitis
VRE: Vancomycin-Resistant Enterococci.

## Funding

None.

## Data availability

The datasets generated and/or analyzed during the current study are available from the corresponding author upon reasonable request.

## Supplementary information

Data must be requested from the corresponding author.

## Declaration of competing interest

The authors declare no conflicts of interest.
